# Oxidative Stress Modulation and Antileishmanial Activity of *Salvinia auriculata*

**DOI:** 10.1002/cbdv.202501027

**Published:** 2025-09-11

**Authors:** Augusto César Rodrigues, Emilha Uzum Papaya, Fernanda da Silva, Carla Cardozo Pinto de Arruda, Edson Lucas dos Santos, Kely de Picoli Souza, Carlos Alexandre Carollo

**Affiliations:** ^1^ Laboratory of Natural Products and Mass Spectrometry (LaPNEM) Federal University of Mato Grosso Do Sul Campo Grande Brazil; ^2^ Research Group on Biotechnology and Bioprospecting Applied to Metabolism (GEBBAM) Federal University of Grande Dourados Dourados Brazil; ^3^ Human Parasitology Laboratory Institute of Biosciences Federal University of Mato Grosso Do Sul Campo Grande Brazil

**Keywords:** antileishmanial efficacy, antioxidant activity, oxidative stress, *Salvinia auriculata*, salvinisides

## Abstract

The genus *Salvinia* has gained attention for its phytoremediation capacity and high levels of bioactive secondary metabolites, positioning it as a potential source of novel therapeutics. Considering the global burden of leishmaniasis and the central role of oxidative stress in disease, this study investigated the antioxidant and antileishmanial properties of the ethanolic extract of *Salvinia auriculata*. Salviniside II, the major metabolite, was isolated, structurally characterized, and quantified. Antioxidant capacity was assessed using in vitro chemical assays (DPPH, ABTS, and FRAP), an ex vivo human erythrocyte model, and in vivo *Caenorhabditis elegans* under juglone‐induced oxidative stress. Antileishmanial activity of the extract and salviniside II was evaluated against *Leishmania amazonensis* and *Leishmania infantum*. The extract showed notable antioxidant and antiparasitic effects, whereas salviniside II alone was less effective at inhibiting parasite growth, suggesting contributions from other compounds or synergistic interactions within the extract. These findings underscore the remarkable accumulation of an underexplored metabolite, salviniside II, in *S. auriculata* and highlight the dual antioxidant and antileishmanial potential of its ethanolic extract. Thus, *S. auriculata* emerges as a promising candidate for developing new therapies targeting oxidative stress‐related conditions and neglected tropical diseases, including leishmaniasis.

## Graphical abstract

Ethanolic extract of *Salvinia auriculata* showed significant antioxidant and antileishmanial activities. Salviniside II, the major metabolite, was isolated, structurally characterized, and evaluated, but exhibited weaker antiparasitic effects when compared to the whole extract.

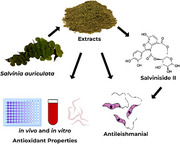

## 1 Introduction

In recent years, neglected tropical diseases have emerged as a critical global health concern, with leishmaniasis representing one of the most significant challenges. An estimated 1.2 million cases of leishmaniasis occur annually, with a mortality rate ranging from 10% to 20% in endemic regions, particularly in tropical areas [1]. In addition, current treatment options are hampered by numerous limitations, including severe side effects, high toxicity, and the rising issue of drug resistance, making them less accessible and effective [2].

Given these challenges, the search for natural, low‐toxicity molecules with therapeutic potential has become increasingly crucial in developing safer and more effective treatment options [3]. Plant‐derived secondary metabolites, especially phenolic compounds, have shown significant promise due to their antioxidant properties and demonstrated anti‐leishmanial effects [4]. These compounds offer a dual benefit: not only can they exert direct antiparasitic effects, but their antioxidant activity may also help modulate host responses, particularly by managing reactive oxygen species (ROS) levels, which are crucial in both the immune response and parasite susceptibility [5].

However, the antioxidant effect in the context of *Leishmania* infection is complex and may exert both beneficial and detrimental outcomes. While antioxidants can mitigate oxidative tissue damage and preserve host immune cell viability, they may also inhibit key leishmanicidal mechanisms dependent on ROS and nitric oxide (NO), which are produced by activated macrophages and neutrophils during the oxidative burst. These molecules are not only cytotoxic to intracellular amastigotes but also essential for the activation of inflammatory pathways such as the NLRP3 inflammasome, which contributes to parasite clearance [6].

Experimental evidence highlights a dual‐edge role for ROS in leishmaniasis. On one hand, ROS produced via the dectin‐1/Syk and TLR2/TLR4 signaling cascades are indispensable for controlling *Leishmania amazonensis* and *Leishmania braziliensis*: pharmacological quenching with *N*‐acetylcysteine (NAC) markedly increases parasite load, confirming that adequate ROS levels are required for parasite restriction. On the other hand, merely raising ROS is not sufficient. Under hyperglycemic conditions, chronically elevated basal ROS fail to clear infection because high glucose suppresses key pattern‐recognition receptors, blunting downstream immune activation. Thus, both the magnitude of ROS and the cellular context in which they arise dictate whether oxidative stress contributes to parasite killing or, conversely, permits persistence [7].

In the context of oxidative stress—an essential factor for both the host's immune defense and the parasite's vulnerability—careful modulation of this stress with antioxidants could serve as a complementary therapeutic approach. Because *Leishmania* parasites depend on a delicate redox balance and are susceptible to oxidative damage, such modulation may enhance treatment efficacy by impairing parasite survival while simultaneously protecting host cells from excessive oxidative injury. Also, these findings highlight the need for further mechanistic studies to clarify the concentration‐dependent and stage‐specific effects of redox‐active compounds in leishmaniasis, particularly regarding their influence on host‐pathogen interactions and immune effector pathways.

The biological relevance of *Leishmania* species varies according to their clinical and pathogenic profiles. *L. amazonensis*, *L. braziliensis*, and *Leishmania infantum* are among the most medically significant species, associated with cutaneous, mucocutaneous, and visceral forms of the disease, respectively. *L. amazonensis* typically causes localized or diffuse cutaneous lesions; *L. braziliensis* is strongly implicated in mucocutaneous leishmaniasis; and *L. infantum* is responsible for visceral leishmaniasis, a systemic and potentially fatal condition if untreated [1]. These clinical manifestations reflect distinct biological adaptations, also evidenced in proteomic analyses. For example, *L. amazonensis* exhibits elevated levels of heat shock proteins (HSPs) and oxidative stress‐related proteins, indicating a robust defense against host‐induced stress. In contrast, *L. braziliensis* shows an abundance of ribosome biogenesis proteins, while *L. infantum* demonstrates a metabolic profile geared toward energy production, consistent with its tropism for internal organs [8].

**FIGURE 1:**
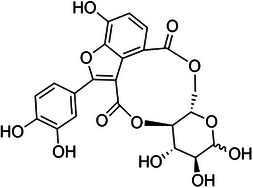
Chemical structure of salviniside II.

The selection of plant species for bioactive studies is typically guided by previous scientific research and reports of their biological activity. In this regard, the aquatic macrophyte *Salvinia auriculata* Aubl. (Salviniaceae), widely distributed throughout South America and adapted to eutrophic and humid environments, stands out. The genus *Salvinia* is of particular interest due to its documented production of bioactive secondary metabolites with antioxidant, anti‐inflammatory, anticancer, and antimicrobial properties [9]. Among these metabolites, salviniside derivatives—exclusive to *Salvinia* species—have garnered interest due to their structural resemblance to macrocyclic lactones like ivermectin. Although salvinisides (Figure 1) possess a smaller 10‐membered ring compared to other macrocyclic compounds, their distinct structural features suggest potential bioactivity, positioning them as promising candidates for further investigation in the treatment of parasitic diseases, such as leishmaniasis [10].

While the structural resemblance to broad‐spectrum antiparasitics such as ivermectin supports the rationale for investigating salvinisides derivatives, the broader biological activity of *S. auriculata* extracts remains underexplored. This study, therefore, focuses on evaluating the antileishmanial and antioxidant properties of the ethanolic extract of *S. auriculata* (EESa). In addition, salviniside II was isolated from the ethanolic extract and its concentration within the extract was determined. This study seeks to enhance the understanding of *S. auriculata* biotechnological potential by evaluating the extract's dual antioxidant and antileishmanial properties, while clarifying the specific contribution of salviniside II to these activities.

## 2 Results and Discussion

The extraction of *S. auriculata* yielded 8.7% of the crude ethanolic extract (CEESa), which corresponds to the initial hydroethanolic extract obtained by percolation of the pulverized plant biomass using a 3:7 (v/v) ethanol:water solvent system. For purification, the CEESa was subjected to a liquid–liquid partition with hexane to remove low‐polarity compounds, resulting in the EESa, a defatted form of CEESa.

Subsequently, the EESa was fractionated by flash chromatography on a silica gel column. The fractionation involved sequential elution with solvent gradients of increasing polarity: first hexane:chloroform (9:1 v/v), then chloroform:ethyl acetate (7:3 v/v), followed by ethyl acetate:methanol (9:1 v/v), and finally 100% methanol. This process yielded four distinct fractions corresponding to different polarity and chemical profiles: Hexane:chloroform fraction (0.4% of total extract), chloroform:ethyl acetate fraction (3.4%), ethyl acetate:methanol fraction (20.4%) (designated as the ethyl acetate fraction of *S. auriculata—*EAcFSa), methanol fraction (27.9%) (designated as the methanolic fraction of *S. auriculata*—MeFSa).

The EAcFSa fraction was further purified by semi‐preparative high‐performance liquid chromatography (HPLC), yielding 32.5 mg of the isolated compound salviniside II.

The predominance of the ethyl acetate:methanol and methanol fractions—together accounting for 48.3% of the total extract—indicates that the major constituents of *S. auriculata* are polar to moderately polar. This profile is typical of secondary metabolites such as flavonoids and phenolic acids, which are frequently associated with antioxidant and antiparasitic activities [11], and it is consistent with reports on aquatic plants, where polar metabolites predominate due to their higher solubility in aqueous environments [12].

Unlike studies on other *Salvinia* species that primarily focus on apolar or partially polar fractions, where *n*‐hexane has been used as the main extraction solvent to recover lipophilic compounds such as steroids [13], this study prioritized polar fractions. Notably, this approach enabled the effective isolation of salviniside II (logP = −0.092), which was enriched to 24.6% in the ethyl acetate/methanol fraction, further demonstrating the efficiency of this fractionation method.

**FIGURE 2:**
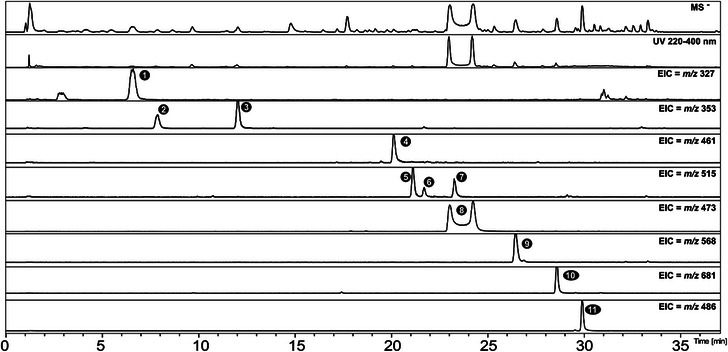
HPLC–DAD–MS chromatogram of the ethanolic extract of *Salvinia auriculata*. The top trace represents the total ion chromatogram (MS detection in negative mode), followed by the UV absorption chromatogram recorded at 220–400 nm. The subsequent traces correspond to extracted ion chromatograms (EIC) for the respective *m*/*z* values, facilitating the visualization and identification of specific compounds. The numbered peaks correspond to the compounds listed in Table 1.

HPLC–diode array detector (DAD)–mass spectrometry (MS/MS) analysis of the EESa extract enabled the identification/annotation of eleven compounds based on UV, MS, and MS/MS data. Peak assignments were performed in both positive and negative ionization modes, with negative mode providing superior spectral quality and peak intensity for compound confirmation (Figure 2). The identified/annotated metabolites are summarized in Table 1.

**TABLE 1:** Identified metabolites in the ethanolic extract of *Salvinia auriculata* (EESa), including their molecular formulas, exact masses, UV absorption maxima, and mass spectrometry data obtained in negative ionization mode (LC–DAD–MS/MS).

Peak	RT (min)	Compound	Molecular formula	Err (ppm)	UV (nm)	Negative mode (*m*/*z*)	
						MS	MS/MS
1	6.6	Unidentified	C_14_H_16_O_9_	−4.5	262, 290	327.0736	173.0485
2	7.8	3‐*O‐E*‐Caffeoylquinic	C_16_H_18_O_9_	−2.8	297, 325	353.0881	191.0548; 179.0277 173.0345; 161.0196
3	12.0	5*‐O‐E*‐Caffeoylquinic	C_16_H_18_O_9_	−2.1	300, 325	353.0885	191.0571; 179.0375; 173.0456; 161.0161
4	20.0	Kaempferol‐*O*‐hexoside	C_21_H_18_O_12_	−1.2	266, 346	461.0731	285.0388
5	21.0	3,4‐Dicaffeoylquinic acid	C_25_H_24_O_12_	−2.1	297, 325	515.1206	335.0795; 191.0562; 179.0344; 173.0477; 161.0250; 155.0380
6	21.6	3,5‐Dicaffeoylquinic acid	C_25_H_24_O_12_	−1.4	298, 329	515.1202	191.0570; 179.0340; 161.0270
7	23.2	4,5‐Dicaffeoylquinic acid	C_25_H_24_O_12_	−0.3	298, 329	515.1197	325.0149; 191.0596; 179.0330; 173.0470; 161.0213
8	22.9	Salviniside II^a^	C_22_H_18_O_12_	−1.3	330	473.0732	413.0533; 369.0572; 353.0353; 325.0349; 311.0285; 285.0387; 267.0297; 253.0496; 241.0521
8	24.1	Salviniside II^a^	C_22_H_18_O_12_	−1.0	330	473.0734	413.0548; 369.0679; 353.0397; 325.0347; 311.0196; 285.0440; 267.0339; 255.0322; 241.0509
9	26.3	Unidentified	C_27_H_22_NO_13_	−4.0	219, 330	568.1119	413.0528; 369.0659; 285.0416; 241.0610
10	28.5	Unidentified	C_37_H_29_O_13_	1.9	220, 330	681.1601	637.1680; 471.0638; 413.0540; 285.0426
11	29.8	Unidentified	C_24_H_40_NO_9_	−4.8	—	486.2731	280.2278

^a^ The peaks listed in the table correspond to anomers of salviniside II, individually reported according to their respective retention times.

Peak 1 exhibits an *m*/*z* 327.0736 [M−H]^−^ compatible with C_14_H_16_O_9_, with a fragment corresponding to shikimic acid at *m*/*z* 173.0485 (C_7_H_9_O_5_^−^). In addition, the UV absorption bands between 260 and 290 nm are consistent with conjugated aromatic systems characteristic of phenolic compounds. However, despite this information, the literature search did not reveal any compatible compounds, and the peak could not be definitively identified.

Peaks 2 and 3, with retention times of 7.8 and 12.0 min, exhibited UV absorption at 300 and 325 nm, characteristic of phenylpropanoid derivatives. These compounds exhibited a deprotonated molecular ion at *m*/*z* 353 [M−H]^−^, with molecular formulas and fragmentation patterns consistent with caffeoylquinic acid derivatives [14]. Based on these data and comparison with authentic standards, Peaks 2 and 3 were identified as 3‐*O*‐E‐caffeoylquinic acid and 5‐*O*‐E‐caffeoylquinic acid, respectively.

Peak 4 exhibited a molecular ion at *m*/*z* 461.0731, with UV absorption bands characteristic of a flavonol skeleton, specifically aligning with kaempferol derivatives, as reported by Enomoto [15]. The presence of a key fragment at *m*/*z* 285 supports its annotation as kaempferol‐*O*‐hexoside. Peaks 6 and 7 were identified as 3,5‐ and 4,5‐dicaffeoylquinic acids, respectively, through comparison with authentic standards. Peak 5 was annotated as 3,4‐dicaffeoylquinic acid, supported by fragment ions at *m*/*z* 173 and the absence of diagnostic ions associated with 1,4‐dicaffeoylquinic acid [14b].

Peak 8 was detected as two distinct anomers with different retention times, both identified as salviniside II in Table 1. The molecular ion at *m*/*z* 473.0732 confirmed the structure as C_22_H_18_O_12_, in agreement with previous reports [9a].

Salviniside II was isolated from an aliquot of the EAcFSa fraction, yielding 32.5 mg from an initial 161 mg of extract (20.2 %). This yield was distributed between the α‐ and β‐anomers, with the β‐anomer predominating (63.3%) over the α‐anomer (36.7%). To investigate their conformational nature, the fractions corresponding to each anomer were individually collected by HPLC and reinjected, consistently yielding two separate peaks. This observation suggests that the anomers exist in a dynamic equilibrium, leading to continuous interconversion during fractionation. Similar behavior has been reported for other systems by Li et al. [9a], posing challenges for isolating each anomer independently.

Nuclear magnetic resonance (NMR) (^1^H and ^13^C) analysis confirmed the identity of salviniside II, yielding spectra consistent with those reported by Li et al. [9a]. Two distinct anomeric proton signals at *δ* = 4.57 (1H, d, *J* = 7.8 Hz, β‐H‐1″) and 5.14 (1H, d, *J* = 3.7 Hz, α‐H‐1″) corresponded to protons at the C‐1 position of glucose. HSQC spectra correlated these signals with carbon resonances at *δ* = 98.6 and 94.4 for the α‐ and β‐anomers, respectively, serving as diagnostic markers of salviniside II anomers.

Salviniside II was the predominant metabolite in the analyzed samples, while salviniside I, previously reported in *Salvinia modesta* [9a], was not detected. In addition, other metabolites typically found in the genus, such as salvinolic acids and natansines, were absent, despite their identification in *Salvinia molesta* and *Salvinia natans* [9a]. While abiotic factors such as light intensity, water quality, and nutrient availability are known to influence secondary metabolite biosynthesis in aquatic plants [16], no seasonal or environmental variation was assessed in this study. Instead, these findings highlight the distinct chemical profile of *S. auriculata*, which may contribute to its unique biological potential. Comparative analyses of other *Salvinia* species under controlled conditions could provide further insights into the metabolic differences observed.

The macrocyclic structure of salviniside II (Figure 1), resembling lactones, and the presence of functional groups within its ring system suggest significant pharmacological potential [10]. Extracts of *S. auriculata* have demonstrated antimicrobial, antioxidant, and selective cytotoxic activity against cancer cells [9a].

Small structural variations, such as those observed in anomers, can markedly influence bioactivity by modulating molecular interactions with biological targets. Anomeric and conformational isomers often exhibit distinct affinities for receptors and enzymes, shaping their pharmacological profiles [9a].

The identification of salviniside II anomers in *S. auriculata* underscores the role of structural diversity in bioactivity. Conformational differences can impact molecular interactions, affecting affinity, specificity, and pharmacological efficacy. Conformational adaptability has been recognized as a key factor in drug discovery [17], and studies have shown that small‐molecule variability can significantly alter functional outcomes [18].

The concentrations of α‐ and β‐salviniside II in the EESa, EAcFSa, and MeFSa fractions were quantified using a calibration curve (*r* = 0.9999), with the peak areas of both anomers summed. This approach ensured consistency in quantification, minimizing variations caused by equilibrium shifts influenced by external factors. The data demonstrated the effectiveness of the salviniside II enrichment process, as evidenced by a nearly sevenfold increase in its content. The concentration rose from 3.69% in the EESa fraction to 24.62% in the EAcFSa fraction and 14.35% in the MeFSA fraction.

The results in Table 2 indicate that EESa exhibited lower antioxidant activity than ascorbic acid (AA) and butylated hydroxytoluene (BHT) in all assays, except for the ferric reducing antioxidant power (FRAP) assay. In this case, EESa demonstrated a reducing power of 374.2 ± 14.6 µg/mL, significantly lower than that of AA (19.0 ± 1.7 µg/mL) but approximately 1.6 times higher than BHT (602.0 ± 43.3 µg/mL), suggesting a moderate ferric‐reducing capacity. These findings suggest that, while EESa possesses antioxidant activity, it is weaker than AA and only moderately more effective than BHT.

**TABLE 2:** Antioxidant activity of EESa and standard antioxidants (AA: ascorbic acid, BHT: butylated hydroxytoluene) in DPPH•, ABTS•^+^, and FRAP assays.

Sample	DPPH•		ABTS•^+^		FRAP	
	IC_50_ (µg/mL)	*A*_max_ (µg/mL)	IC_50_ (µg/mL)	*A*_max_ (µg/mL)	EC_50_ (µg/mL)	*A*_max_ (µg/mL)
AA	5.3 ± 0.8	50	3.2 ± 0.3	5	19.0 ± 1.7	10
BHT	5.9 ± 1.5	50	10.1 ± 2.8	50	602.0 ± 43.3	500
EESa	94.6 ± 9.9	500	50.3 ± 0.06	>100	374.2 ± 14.6	100

*Note*: IC_50_ represents the concentration required to scavenge 50% of DPPH• and ABTS•^+^ radicals, while EC_50_ corresponds to the concentration needed to reduce Fe^3+^ to Fe^2+^. *A*_max_ indicates the maximum activity of the extract. Data are expressed as mean ± mean standard error (MSE).

This evaluation served as an initial screening to determine EESa potential for further studies. Phenolic compounds, as caffeoylquinic acids and flavonoids, are known for their radical scavenging properties [19], and variations in IC_50_ values among plant extracts highlight the influence of phytochemical composition on antioxidant efficacy [20].

At this stage, fractionation and isolated compounds were not tested, as the primary aim was to evaluate the antioxidant capacity of the crude extract, whose chemical composition was determined (Table 1). In addition, the high concentration of salviniside II in the fraction limited further testing, as its potential influence on the overall antioxidant profile could overshadow the effects of other bioactive compounds. The increasing demand for natural antioxidants, due to concerns over synthetic alternatives [21], underscores the relevance of these findings. In this context, the next analysis will assess EESa antioxidant potential in human erythrocyte models to further elucidate its biological relevance.

The hemolysis assay (Figure S1) demonstrated a dose‐dependent effect of EESa on human erythrocytes. While concentrations up to 250 µg/mL induced minimal hemolysis, a significant increase was observed at 500 µg/mL, suggesting potential cytotoxic effects at higher doses due to membrane destabilization. In comparison, AA exhibited a protective effect at lower concentrations but also induced moderate hemolysis at 250 µg/mL. These findings suggest that EESa helps maintain erythrocyte integrity at low to moderate concentrations but may compromise membrane stability at higher doses, warranting further investigation into its bioactive components and mechanisms of action.

**FIGURE 3:**
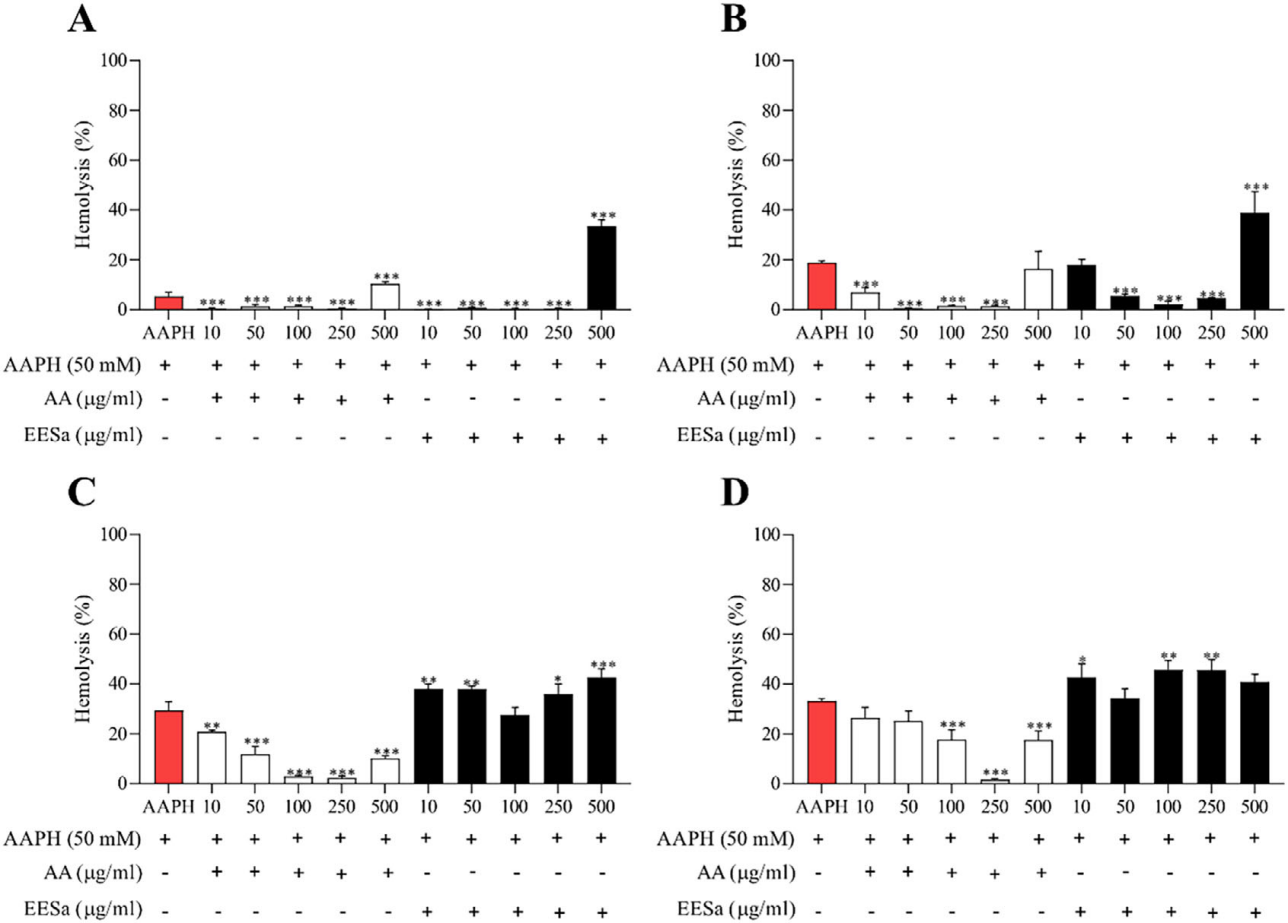
Effect of EESa (10–500 µg/mL) and ascorbic acid (AA, standard antioxidant) on AAPH‐induced hemolysis (50 mM) at different incubation times: (A) 1 h, (B) 2 h, (C) 3 h, and (D) 4 h. Data are presented as mean ± standard error of the mean (SEM) from two independent experiments performed in triplicate. ***, **, and * indicate statistically significant differences (*p* < 0.01, *p* < 0.05, *p* < 0.1, respectively) compared to the AAPH‐treated group.

EESa exhibited a time‐ and concentration‐dependent effect on AAPH‐induced hemolysis, as shown in Figure 3, displaying protective activity at 10–250 µg/mL, particularly in early incubation periods (1–2 h). However, at 500 µg/mL, hemolysis increased significantly across all time points, with the most pronounced effect observed after 3 and 4 h. These findings suggest that while EESa exerts antioxidant properties at lower concentrations and shorter exposure times, prolonged incubation and higher doses may lead to membrane destabilization, warranting further investigation into its mechanisms and potential applications.

The observed hemolytic activity of EESa at higher concentrations aligns with the dual role of phenolic compounds as both antioxidants and pro‐oxidants, as highlighted by Rajashekar [22]. This phenomenon is particularly relevant in oxidative environments, where phenolic compounds can undergo redox cycling, generating ROS that may contribute to cellular damage instead of protection. The pro‐oxidant effect at elevated concentrations suggests that, while EESa may confer antioxidant benefits at lower doses, its application must be carefully considered to avoid cytotoxic effects. Future studies should investigate the mechanistic basis of this biphasic response, particularly in physiological settings where oxidative stress and redox balance are critical determinants of cellular homeostasis.

**FIGURE 4:**
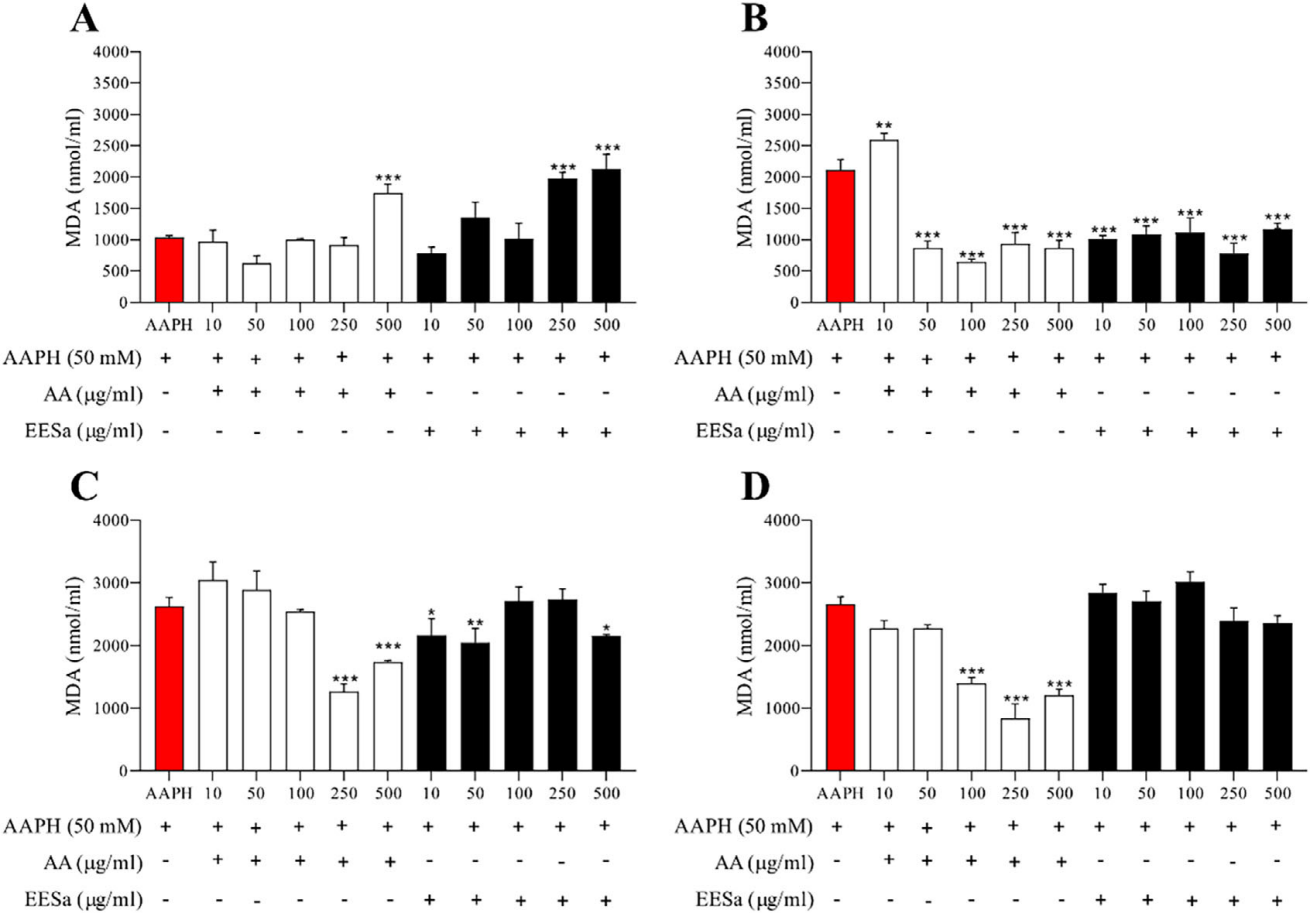
Effect of EESa (10–500 µg/mL) and ascorbic acid (AA, standard antioxidant) on AAPH‐induced lipid peroxidation, measured by malondialdehyde (MDA) formation (nmol/mL) at different incubation periods: (A) 1 h, (B) 2 h, (C) 3 h, and (D) 4 h. Data are presented as mean ± standard error of the mean (SEM) from two independent experiments performed in triplicate. ***, **, and * indicate statistically significant differences (*p* < 0.01, *p* < 0.05, *p* < 0.1, respectively) compared to the AAPH‐treated group.

Based on Figure 4, the effect of EESa on AAPH‐induced lipid peroxidation, measured by malondialdehyde (MDA) formation, also exhibited a time‐ and concentration‐dependent pattern. At 1 h (Figure 4A), neither EESa nor AA significantly reduced MDA levels, suggesting that both antioxidants required more time to exert their protective effects. At 2 h (Figure 4B), AA and EESa demonstrated comparable inhibition of lipid peroxidation, with EESa at low concentration (10 µg/mL) showing greater protection than AA, reinforcing its potential as a radical scavenger in early oxidative events at low concentrations. After 3 h (Figure 4C), AA maintained its protective role at 250 and 500 µg/mL, while EESa continued to provide some protection at lower concentrations (10 and 50 µg/mL), but its efficacy diminished at 100 µg/mL and above. This suggests that while EESa retains some antioxidant capacity in early lipid peroxidation stages, its protective effect weakens over time, particularly at higher concentrations, where oxidative instability may lead to redox cycling and pro‐oxidant activity. By 4 h (Figure 4D), AA continued to reduce MDA formation at higher concentrations, while EESa completely lost its antioxidant effect, with MDA levels returning to those observed in the AAPH control.

These findings align with previous studies on phenolic‐rich extracts, which report that antioxidants can exhibit biphasic behavior, acting as protective agents at lower concentrations but promoting oxidative damage at higher doses or prolonged exposure [23].

The observed loss of efficacy over time may be due to redox cycling, oxidative degradation of active compounds, or depletion of antioxidant capacity [24]. This emphasizes the importance of considering the temporal stability and redox dynamics of natural antioxidants when evaluating their potential biological applications.

The results presented in Figure S2 indicate that EESa did not exhibit significant toxicity in *Caenorhabditis elegans* across the tested concentrations, as no substantial reduction in viability was observed compared to the control group. These findings are essential for validating the subsequent oxidative stress assays, ensuring that any protective or detrimental effects observed in stress‐induced models are not confounded by intrinsic toxicity. Previous studies have emphasized the relevance of *C. elegans* as a model for evaluating the antioxidant effects of phytochemicals, particularly due to its conserved oxidative stress response pathways [25].

**FIGURE 5:**
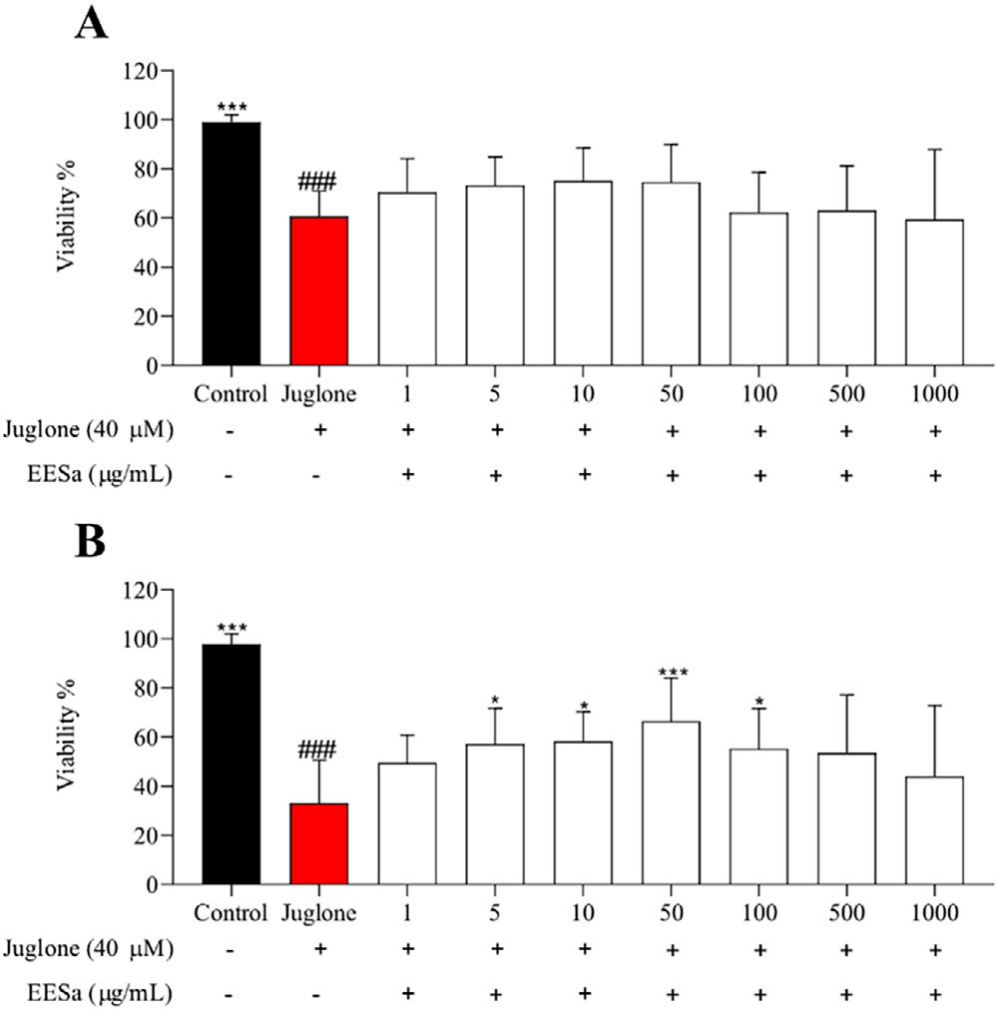
Effect of EESa on *Caenorhabditis elegans* survival under juglone‐induced oxidative stress. Viability (%) of *C. elegans* exposed to M9 medium (control) and juglone (40 µM) alone or co‐treated with different concentrations of EESa (1–1000 µg/mL) after 24 h (A) and 48 h (B) of incubation. Data are presented as mean ± SEM from three independent experiments conducted in triplicate. ^###^ indicates a significant reduction in viability compared to the control group (*p* < 0.001); ***, **, and * indicate significant differences (*p* < 0.001, *p* < 0.01, and *p* < 0.05, respectively) compared to the juglone‐treated group.

The results presented in Figure 5 demonstrate that EESa exhibited a concentration‐dependent effect on the viability of *C. elegans* under juglone‐induced oxidative stress. At lower concentrations (10–100 µg/mL), EESa significantly improved survival compared to the juglone‐treated control, suggesting a protective effect against oxidative damage. This aligns with previous findings on the antioxidant potential of plant‐derived polyphenols, which have been shown to enhance stress resistance and promote longevity through oxidative stress mitigation mechanisms [26].

However, at the highest concentration tested (500–1000 µg/mL), EESa failed to confer protection, and viability was comparable to the juglone‐only group, indicating a potential pro‐oxidant effect at elevated doses. This biphasic response is consistent with reports that certain polyphenols exhibit hormetic properties, where low concentrations activate protective cellular pathways, whereas higher doses induce oxidative stress and cytotoxicity [22].

The observed protective effects at intermediate concentrations may be attributed to the modulation of stress response pathways, such as DAF‐16/FOXO or SKN‐1/Nrf2, which have been implicated in polyphenol‐mediated stress resistance and longevity in *C. elegans* [26a]. These findings pave the way for further investigations into the precise molecular mechanisms underlying EESa antioxidant properties, including its potential role in modulating oxidative stress‐related signaling pathways and its long‐term impact on organismal health and survival.

The results presented in Table 3 indicate the antileishmanial activity of the ethanolic extract (EESa), its EAcFSa, and its major metabolite, salviniside II, against *L. amazonensis* and *L. infantum*. EESa exhibited moderate activity against *L. amazonensis* (IC_50_ = 11.69 µg/mL) but was less effective against *L. infantum* (41.39 µg/mL). Fractionation into EAcFSa did not enhance activity, as its IC_50_ remained at 50 µg/mL for both species, suggesting that bioactive compounds were either present at lower concentrations or required synergistic interactions lost upon fractionation.

**TABLE 3:** IC_50_ values (µg/mL) of EESa, EAcFSa, salviniside II, pentamidine, and amphotericin B against *Leishmania amazonensis* and *Leishmania infantum*.

Sample	*L. amazonensis* IC_50_ (µg/mL)	*L. infantum* IC_50_ (µg/mL)
EESa	11.69	41.39
EAcFSa	50.00	50.00
Salviniside II	16.89	50.00
Pentamidine	1.58	3.03
Amphotericin B	0.19	0.14

Salviniside II, the major metabolite from EAcFSa, displayed greater activity than its fraction against *L. amazonensis* (16.89 µg/mL) but was ineffective against *L. infantum* (50 µg/mL), reinforcing the possibility that other compounds in the crude extract may enhance the antileishmanial effect through synergistic interactions.

The superior performance observed for the EESa, compared to the isolated salviniside II, may be attributed to the presence of synergistic effects among the various secondary metabolites present in the extract matrix. As widely reported in the literature, the biological activity of plant extracts often results from the combined action of multiple compounds, such as phenolics, flavonoids, and other classes of metabolites, which may act synergistically or additively to enhance antiparasitic effects [27].

In this context, the greater effectiveness of the whole extract suggests that its activity is not exclusively related to a single active compound, but rather to the interaction among different substances that may facilitate cellular target penetration, modulate the bioavailability of active constituents, or help overcome parasite resistance mechanisms.

In addition, the use of ethanol as a solvent favors the acquisition of a broader chemical profile, capable of extracting a diverse range of metabolites with varying polarities. Even at low concentrations, these compounds may exert complementary or modulatory effects, thereby enhancing the overall biological activity of the extract. These findings highlight the importance of strategies that focus on the evaluation of fractions and combinations of plant‐derived compounds, as well as the development of formulations that preserve the natural phytochemical complexity of extracts. Such approaches appear promising for expanding the therapeutic potential of natural products, particularly in the context of neglected diseases such as leishmaniasis [27].

This study also observed that *L. amazonensis* exhibits greater sensitivity to the tested treatments, including plant extracts, isolated metabolites, and reference drugs such as amphotericin B and pentamidine, when compared to *L. infantum*. This difference may be attributed to physiological and biochemical factors between the species, such as membrane composition, permeability, expression of metabolizing enzymes, and resistance mechanisms. *L. infantum*, in turn, appears to possess adaptations that confer greater tolerance to chemical stress, possibly associated with its evolutionary history and the immunological environment of the host [27a].

In addition, proteomic data suggest that these variations are also related to species‐specific metabolic and functional networks. *L. amazonensis* shows a higher abundance of stress‐response‐related proteins, such as HSPs and redox components, which may modulate its drug responsiveness. *L. braziliensis* is characterized by intense ribosomal expression, while *L. infantum* displays elevated levels of proteins associated with energy metabolism, such as succinyl‐CoA ligase, indicating a potentially greater metabolic robustness under treatment conditions [8]. These differences highlight the importance of considering interspecies variability in the development of more effective therapies against leishmaniasis.

A limitation of the present study is that the antileishmanial activity was evaluated only in promastigote forms. Although this model is commonly employed as an initial screening tool in natural product studies, it does not fully reflect the clinical relevance of intracellular amastigotes [28]. Therefore, the results reported here should be considered preliminary. Future studies from our group will incorporate axenic and intramacrophage amastigote assays to provide a more comprehensive evaluation of the therapeutic potential of *S. auriculata* extracts and salviniside II.

The antileishmanial activity observed for salviniside II and EESa represents the first report of such effects within the *Salvinia* genus. Given that Salviniside II possesses a macrolide structure that resembles ivermectin, it is plausible that it shares mechanistic features with this compound. Ivermectin has been shown to induce plasma membrane rigidity in *L. amazonensis* promastigotes through oxidative stress, leading to alterations in membrane permeability and ionic balance, which compromise parasite viability [29].

These observations reinforce the hypothesis that the antileishmanial effects observed for EESa and salviniside II may involve redox‐mediated mechanisms. Although the antioxidant capacity of these compounds may confer cytoprotective benefits by mitigating oxidative damage to host tissues, their pro‐oxidant activity at higher concentrations appears to play a crucial role in compromising parasite viability. In the context of *Leishmania* infection, ROS and NO are essential mediators of the host's leishmanicidal arsenal, particularly in macrophages and neutrophils, where their production is tightly regulated to ensure parasite clearance without inducing excessive host damage [6a]. ROS generation can activate downstream immune pathways, including inflammasome assembly, as demonstrated in macrophages infected with *L. amazonensis*, where ROS produced via dectin‐1/Syk signaling were shown to trigger NLRP3 inflammasome activation and IL‐1β release, ultimately limiting parasite replication [6b].

Conversely, dysregulated oxidative responses may hinder host defense mechanisms. Under hyperglycemic conditions, despite elevated basal ROS levels, macrophages fail to mount an effective oxidative response upon *L. braziliensis* infection, resulting in reduced expression of TLR2 and TLR4 and impaired pathogen recognition and elimination [7].

Furthermore, chemical inhibition of ROS with NAC increases parasite burden, underscoring the importance of ROS in parasite control. Taken together, these findings suggest that oxidative stress, when adequately regulated, can be exploited therapeutically to enhance parasite susceptibility, particularly through mechanisms that destabilize parasite membranes or modulate critical redox‐sensitive signaling pathways. The dual activity of salviniside II, combining antioxidant and pro‐oxidant properties depending on the concentration, highlights the need for further investigation into its specific mechanism of action. Clarifying whether its antileishmanial effects arise from direct membrane disruption, mitochondrial impairment, or modulation of host redox signaling pathways such as dectin‐1/Syk/NLRP3 or TLR2/4, will be essential for the rational development of redox‐active therapeutics against leishmaniasis.

Similarly, our findings suggest that the pro‐oxidant effects observed at higher concentrations of EESa may contribute to its leishmanicidal activity, supporting the idea that oxidative stress plays a role in parasite clearance. The modulation of oxidative and immune responses in *Leishmania* infection has been previously reported as a key mechanism in the action of several natural and synthetic compounds, highlighting the importance of redox homeostasis in parasite survival [30]. Further investigations are required to determine whether salviniside II exerts its activity through direct interactions with the parasite membrane or by triggering oxidative stress pathways similar to those of ivermectin.

## 3 Conclusion

This study provides the first report of the antioxidant and antileishmanial activities of *S. auriculata* and its major metabolite, salviniside II. The ethanolic extract (EESa) exhibited a biphasic redox profile, demonstrating antioxidant effects at moderate concentrations but shifting towards pro‐oxidant activity at higher doses in both chemical assays (DPPH, ABTS, and FRAP) and biological models, including human erythrocytes and *C. elegans* under juglone‐induced oxidative stress.

EESa also displayed antileishmanial activity, with greater potency against *L. amazonensis* than *L. infantum*, while its enriched fraction (EAcFSa) and salviniside II showed reduced efficacy, suggesting a synergistic effect among multiple bioactive compounds. Although salviniside II was identified as the principal metabolite, its isolated activity was moderate, reinforcing the need to explore its structural and functional role in redox modulation and parasite inhibition.

The dual redox behavior of EESa and its potential role in oxidative stress‐related mechanisms align with reported antiparasitic strategies that exploit ROS accumulation and membrane destabilization. Future studies should prioritize mechanistic investigations and in vivo validation of *S. auriculata* metabolites, as well as further evaluation against amastigote forms, including intramacrophage infection models, to confirm the clinical relevance of the preliminary promastigote results reported here in the treatment of leishmaniasis and oxidative stress‐related diseases.

## 4 Experimental

### 4.1 Collection and Extraction of Plant Material

*S. auriculata* was collected from Lagoa Comprida Municipal Natural Park, located in the hydrographic basin of the Aquidauana River, Aquidauana, Mato Grosso do Sul, Brazil (20°27′48.0″ S, 55°46′40.2″ W). This location is situated between the ecotone Cerrado and Pantanal. The plants were dried in an oven with air circulation at 50°C for 72 h until a constant weight was achieved (700 g). This study was conducted in compliance with Brazilian biodiversity regulations, and access to genetic resources was registered under SISGEN authorization A15EB96. Reference voucher specimens are deposited at the Herbarium of the Federal University of Mato Grosso do Sul (CGMS/UFMS) under accession numbers CGMS 53839 and CGMS 1083807.

The hydroethanolic extract (3:7 v/v) was obtained by percolation from pulverized biomass. Extraction occurred over 72 h with alternating flow rates. For the first 48 h, the flow rate was maintained at 30 drops per minute, followed by 24 h at 20 drops per minute. The resulting extract was concentrated by rotary evaporation, followed by lyophilization, and was designated as the CEESa.

### 4.2 Clean‐Up and Fractionation of EESa

The CEESa was first cleaned through liquid–liquid partitioning with hexane to remove nonpolar impurities, resulting in the ethanolic extract fraction (EESa). The cleaned extract (EESa) was then fractionated on a silica gel column using flash chromatography, separating compounds based on polarity. The column was eluted with a gradient of hexane:chloroform (9:1 v/v); chloroform:ethyl acetate (7:3 v/v); ethyl acetate:methanol (9:1 v/v); and finally, methanol (100%) to elute the most polar constituents. The ethyl acetate:methanol fraction was designated as the EAcFSa, and the methanol fraction as the MeFSa.

### 4.3 LC–DAD–MS

The extract EESa was prepared at a concentration of 1 mg/mL in methanol:water (7:3, v/v), then filtered through a PTFE filter (Millex 0.22 µm × 13 mm, Millipore). Subsequently, 3 µL of the filtered solution was injected into a Shimadzu Prominence UFLC system coupled with a DAD and a MicrOTOF‐Q III mass spectrometer (Bruker Daltonics). The chromatographic column used was a Kinetex C18 column (2.6 µm, 150 × 2.1 mm, Phenomenex). The flow rate was set to 0.3 mL/min, and the oven temperature to 50°C. The mobile phase comprised 0.1% formic acid (v/v) in both water (Solvent A) and acetonitrile (Solvent B), utilizing a gradient elution profile as follows: 0–2 min: 3% B; 2–25 min: 3%–25% B; 25–40 min: 25%–80% B; 40–43 min: 80% B; 43–48 min: column washing and re‐equilibration. The analyses were carried out in both negative and positive ion modes. Nitrogen gas was used as both a nebulizer gas (at 4 bar) and a dry gas (at 9 L/min). The capillary voltage was set to 2.5 kV.

### 4.4 Isolation and Characterization of Salviniside II

For the isolation of salviniside II, a portion of the EAcFSa fraction was utilized. An optimized method was developed using a Shimadzu HPLC system equipped with a semi‐preparative C18 column (250 mm × 10 mm, 5 µm particle size, Shimadzu). Method parameters were set as follows: injection volume of 500 µL, flow rate of 9 mL/min, and the retention time for salviniside II was 15 min. The mobile phase consisted of water (Solvent A) and methanol (Solvent B), with a gradient elution profile of 0–15 min: 50%–80% B, followed by 15–18 min: 50% B. The EAcFSa fraction concentration was 20 mg/mL for this procedure.

For the characterization of salviniside II, in addition to HPLC–DAD–MS/MS analysis, an aliquot of 10 mg of the isolated compound was dissolved in 0.6 mL of MeOD/D_2_O (7:3 v/v) for NMR analysis. The NMR spectra were recorded on a Bruker DPX‐500 instrument (^1^H: 500 MHz; ^13^C: 125 MHz), with tetramethylsilane (TMS) used as the internal reference. Chemical shifts (*δ*) are reported in parts per million (ppm), while coupling constants (*J*) are expressed in Hertz (Hz).

### 4.5 Quantification of Salviniside II

The quantification of salviniside II in *S. auriculata* extracts and fractions was performed using LC–DAD at 320 nm. A quantification curve was generated using an HPLC–DAD system (Shimadzu UFLC). The chromatographic conditions, including the column and gradient, were the same as those described in the LC–DAD–MS method. A calibration curve was prepared by performing serial dilutions of a 1 mg/mL salviniside II stock solution, resulting in successive concentrations of 250, 125, 62.5, 31.3, 15.6, 7.8, and 3.9 µg/mL.

To ensure accuracy, the coefficient of determination (*r*) was evaluated, and data linearity was verified using a 95% confidence interval (*p* > 0.05). The linearity criterion was a ±5% variation around the median, ensuring reliable quantification of salviniside II in the extract [31].

### 4.6 DPPH Radical Scavenging Assay

The DPPH• radical scavenging capacity was assessed following Rocha et al. [32]. In this assay, 200 µL of extract was mixed with 1800 µL of 0.11 mM DPPH• in 80% ethanol and incubated at room temperature in the dark for 30 min. Absorbance was measured at 517 nm. AA and BHT were used as reference antioxidants, with 80% ethanol as the control. The experiment was conducted in duplicate, with each run performed in triplicate. The inhibition percentage was calculated relative to the control using the following equation:

$$\begin{array}{ccc} & & \mathrm{DPPH}•\mathrm{scavenging}\;\mathrm{activity}\left(\%\right)\\ & & \;=\left(1\;-\;\mathrm{sample}\;\mathrm{Abs}/\mathrm{control}\;\mathrm{Abs}\right)\;\times \;100 \end{array}$$

### 4.7 ABTS Radical Scavenging Assay

The ABTS•^+^ radical scavenging capacity was determined following Rocha et al. [32]. ABTS•^+^ was prepared by mixing 5 mL of 7 mM ABTS•^+^ with 88 µL of 140 mM potassium persulfate and incubating in the dark for 12–16 h. The solution was diluted in ethanol to an absorbance of 0.70 ± 0.05 at 734 nm. Then, 20 µL of extract was added to 1980 µL of the ABTS•^+^ solution and incubated for 6 min. Absorbance was measured at 734 nm. AA and BHT were used as positive controls. The experiment was conducted in duplicate across two independent assays, with the inhibition percentage calculated using the following equation:

$$\begin{array}{ccc} & & \mathrm{Inhibition}\;\mathrm{of}\mathrm{ABTS}{•}^{+}\left(\%\right)\\ & & \;=\left[1\;-\;\left(\mathrm{Abs}\;\mathrm{sample}/\mathrm{Abs}\;\mathrm{control}\right)\right]\;\times \;100 \end{array}$$

### 4.8 The FRAP Assay

The FRAP assay was adapted from Rocha et al. [32]. In the experiment, 20 µL of extract (1–1000 µg/mL) was mixed with 280 µL of FRAP reagent, which contained TPTZ, FeCl_3_·6H_2_O, and acetate buffer (pH 3.6). The mixture was incubated at 37°C for 30 min, and absorbance was measured at 492 nm. AA, BHT, and ammonium iron sulfate served as standards. Two independent experiments were conducted in triplicate, and EC_50_ values were calculated using regression equations.

### 4.9 Antioxidant Evaluation Using Human Erythrocyte Models

#### 4.9.1 Preparation of Erythrocyte Suspension

Following approval from the Research Ethics Committee of the Federal University of Grande Dourados (UFGD; approval number 6.166.432), and after obtaining prior informed written consent from all volunteers, blood samples from healthy donors were collected in sodium citrate tubes, centrifuged, and washed with 0.9% NaCl to obtain a 10% erythrocyte suspension. The assays using human erythrocyte models were conducted following the protocol outlined by Rocha et al. [32].

#### 4.9.2 Hemolysis and AAPH‐Induced Oxidative Hemolysis Assay

Following Campos et al. [33]. A total of 250 µL of a 10% erythrocyte suspension was pre‐incubated with 250 µL of EESa (10–500 µg/mL) or AA at 37°C for 30 min. Hemolysis was induced by adding 500 µL of 50 mM AAPH. Controls included saline, solvent (1.6% ethanol), and total hemolysis (distilled water). After incubation (60–240 min), samples were centrifuged, and 200 µL of supernatant was mixed with 1800 µL of 0.9% NaCl. Absorbance was measured at 540 nm. Two independent experiments were performed in triplicate, and hemolysis was calculated as:

$$\mathrm{Total}\;\mathrm{hemolysis}\left(\%\right)=\left(A/B\right)\;\times \;100$$

#### 4.9.3 MDA Assay

Following the adapted protocol of Campos et al. [33] a 10% erythrocyte suspension was used to assess EESa's effect on lipid peroxidation. Erythrocytes were pre‐incubated with EESa (10–500 µg/mL) or AA at 37°C for 30 min. After adding 500 µL of 50 mM AAPH, samples were incubated for 60–240 min. MDA was measured by reacting 500 µL of the supernatant with 1 mL of thiobarbituric acid (TBA), followed by incubation at 96°C for 45 min and centrifugation. Absorbance was measured at 532 nm. The experiment was conducted in triplicate, and MDA levels were expressed in nmol/mL using the formula:

$$\begin{array}{ccc} & & \mathrm{MDA}\left(\mathrm{nmol}/\mathrm{mL}\right)=\mathrm{sample}\;\mathrm{absorbance}\\ & & \;\times \left(20\;\times \;220.32/\mathrm{MDA}\;\mathrm{standard}\;\mathrm{absorbance}\right) \end{array}$$

#### 4.9.4 Toxicity Assays in *C. elegans*

The N2 strain of *C. elegans*, obtained from the Caenorhabditis Genetics Center, was cultured on Nematode Growth Medium (NGM) agar with *Escherichia coli* OP50. L4‐stage individuals, representing the young adult phase, were selected for the assays. For synchronization, nematodes and eggs were washed from the plates, treated with sodium hypochlorite (2%) and NaOH (5 M) to release the eggs, which were then transferred to fresh NGM plates. Plates were incubated at 20°C for 48 h until the nematodes reached the L4 stage.

An acute toxicity assay was performed on L4‐stage *C. elegans* to evaluate the in vivo toxicity of CEESa extract, following Vilasboas‐Campos et al. [34] with adaptations. In a 96‐well plate, 10–20 nematodes were placed in 100 µL of M9 medium, followed by the addition of 100 µL of CEESa at concentrations of 1–1000 µg/mL. Positive and solvent controls were also included. Plates were incubated at 20°C for 24 and 48 h. Nematode viability was assessed under a stereoscopic microscope, and results were expressed as the percentage of live nematodes, calculated by:

$$\mathrm{Nematode}\;\mathrm{viability}\left(\%\right)=\left(\mathrm{Ni}\;\times \;100\right)/\mathrm{Nf}$$

#### 4.9.5 Juglone‐Induced Oxidative Stress Assay in *C. elegans*

Following Vilasboas‐Campos et al. [34] *C. elegans* at the L4 stage were exposed to juglone (40 µM) to induce oxidative stress. Nematodes were transferred to 96‐well plates (10 per well) containing 100 µL of M9 buffer. Extracts (1–1000 µg/mL) or M9 (control) were added. After 1 h, 50 µL of juglone was added to all groups except the untreated control. Viability was assessed at 24‐ and 48‐h using touch sensitivity. Two independent experiments were performed in triplicate, and results were expressed as the percentage of live nematodes:

$$\mathrm{Nematode}\;\mathrm{viability}\left(\%\right)=\left(\mathrm{Ni}\;\times \;100\right)/\mathrm{Nf}$$

#### 4.9.6 Anti‐Leishmania Assay

*L. amazonensis* (IFLA/BR/1967/PH8) and *L. infantum* (MHOM/BR/2022/LV072_22) promastigotes were cultured in Schneider's insect medium supplemented with fetal bovine serum, penicillin, and streptomycin. Promastigotes (10^6^ parasites/mL) were incubated with test samples (0.78–50 µg/mL) in a 96‐well plate at 26°C for 72 h. Viability was assessed using MTT, and IC_50_ values were calculated with GraphPad Prism 8.0. Amphotericin B and pentamidine were used as positive controls, and Schneider medium as the negative control.

#### 4.9.7 Statistical Analysis of Antioxidant and Toxicity Assays

Results were expressed as mean ± SEM. IC_50_ and EC_50_ values were determined through non‐linear regression. Group comparisons were made using ANOVA followed by Dunnett's test. All analyses were performed in GraphPad Prism 8.0, with *p* < 0.05 considered statistically significant.

## Author Contributions

A.C.R. and C.A.C. designed the study and wrote the manuscript. E.U.P., F.S., E.L.S., C.C.P.A., and K.P.S. performed the experiments, data analysis, and contributed to the construction of figures and tables. C.A.C., E.L.S., and K.P.S. reviewed the manuscript and provided critical revisions. All authors read and approved the final manuscript.

## Conflicts of Interest

The authors declare no conflicts of interest.

## Supporting information




**Supporting File 1**: cbdv70496‐sup‐0001‐SuppMat.pdf

## Data Availability

The authors have nothing to report.
