# Supplementary material for: Oxidative Stress Modulation and Antileishmanial Activity of Salvinia auriculata
Source: Chem Biodivers. 2025 Sep 11;22(12):e01027. doi: 10.1002/cbdv.202501027 (PMC12716024; doi:10.1002/cbdv.202501027)
Supplement: Supplementary file 1 — Supporting File 1: cbdv70496‐sup‐0001‐SuppMat.pdf [file CBDV-22-e01027-s001.pdf]

Oxidative Stress Modulation and Antileishmanial Activity of *Salvinia auriculata*

Augusto César Rodrigues[1], Emilha Uzum Papaya[2], Fernanda da Silva[3], Carla Cardozo Pinto de Arruda[3], Edson Lucas dos Santos[2], Kely de Picoli Souza[2], Carlos Alexandre Carollo[1,*]

1 Laboratory of Natural Products and Mass Spectrometry (LaPNEM), Federal University of Mato Grosso do Sul, Campo Grande, MS, Brazil

2 Research Group on Biotechnology and Bioprospecting Applied to Metabolism (GEBBAM), Federal University of Grande Dourados, Dourados, MS, Brazil

3 Human Parasitology Laboratory, Institute of Biosciences, Federal University of Mato Grosso do Sul, Campo Grande, MS, Brazil.

*Correspondence:
Carlos Alexandre Carollo, Laboratory of Natural Products and Mass Spectrometry, Faculty of Pharmaceutical Sciences, Food, and Nutrition, Federal University of Mato Grosso do Sul, Campo Grande, MS, 79070900, Brazil
Email: carlos.carollo@ufms.br

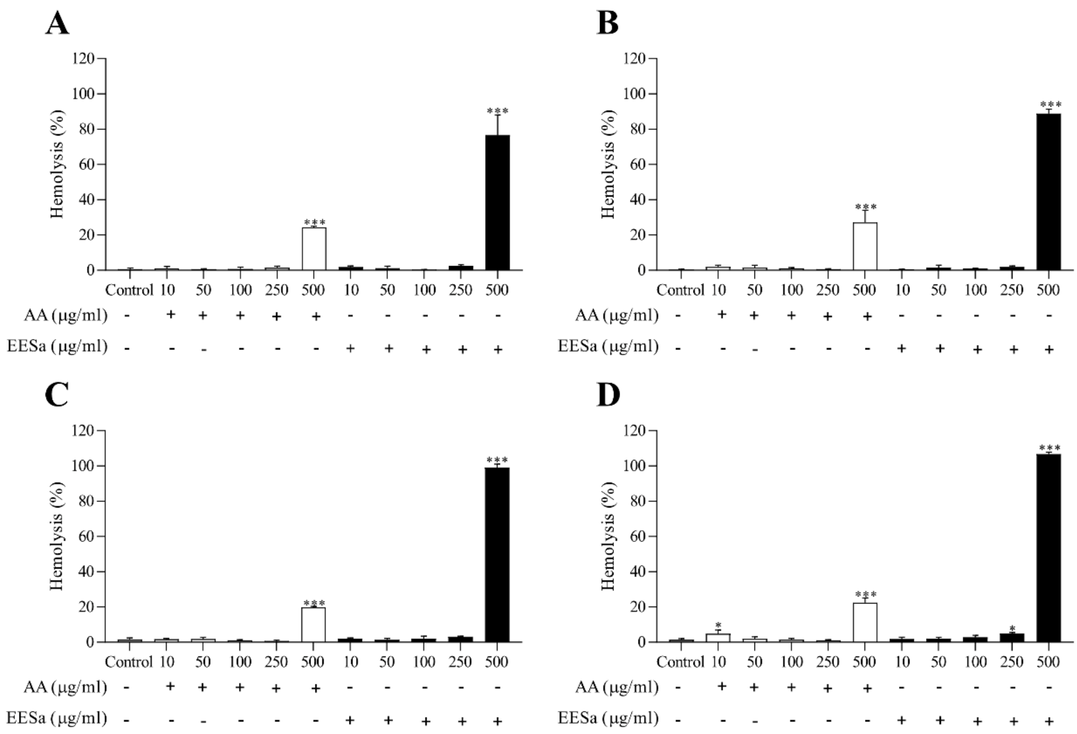

Figure S1. Hemolytic assay, effect of varied concentrations (10 - 500 µg/mL) of Ethanolic Extract of *Salvinia auriculata* (EESa) and Ascorbic Acid (AA) at different incubation intervals [(A) 1 hour, (B) 2 hours, (C) 3 hours, (D) 4 hours]. The results are presented as the mean ± Standard Error of the Mean (SEM) from two independent experiments conducted in triplicate. ***, **, * represent statistically significant results ($P < 0.1$; $P < 0.05$; $P < 0.01$, respectively) when comparing groups treated with EESa or AA to the control group. Control represents saline control, where erythrocytes were incubated only with 0.9% NaCl solution.

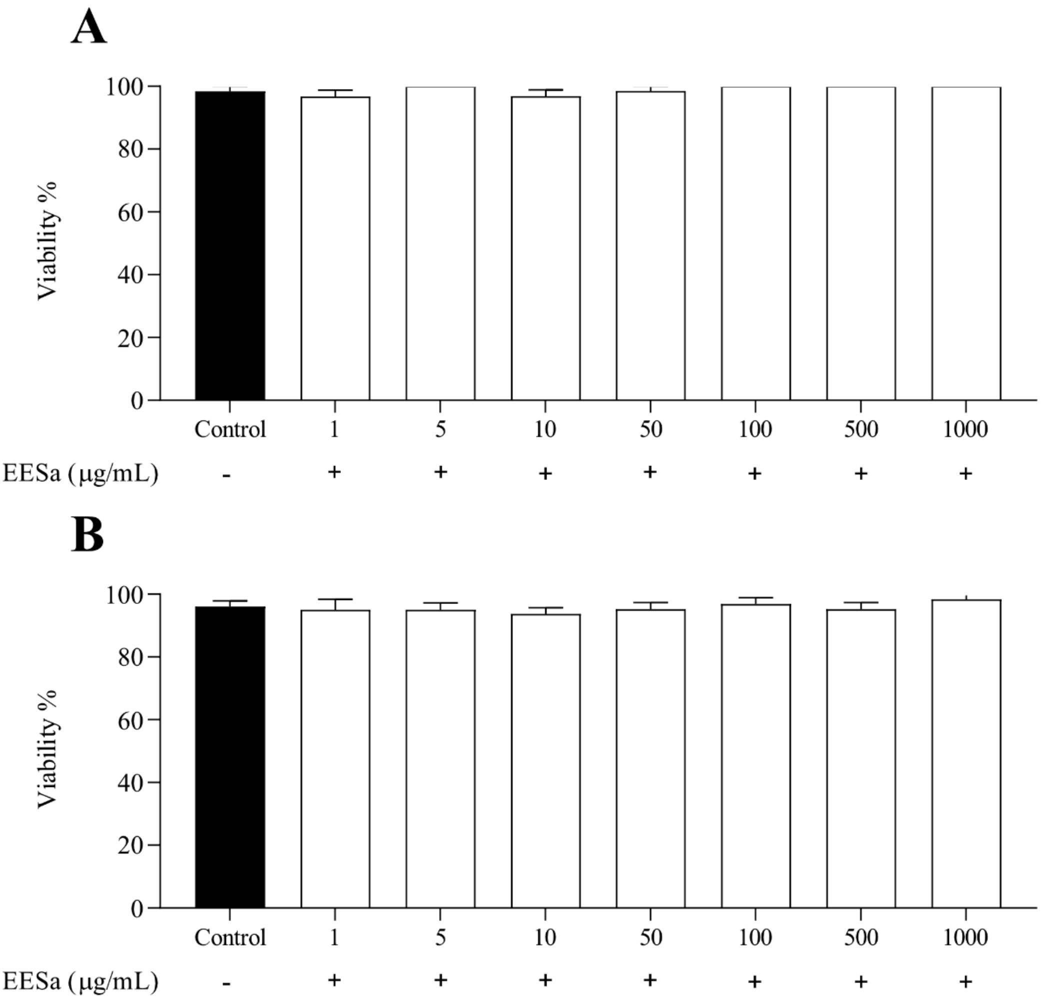

Figure S2. Toxicity assessment in the *C. elegans* model. Viability of *C. elegans* (%) following exposure to M9 medium (Control) and various concentrations of EESa (1–1000 µg/mL) after 24 hours (A) and 48 hours (B) of incubation. Data are presented as mean ± SEM from two independent experiments, each conducted in triplicate.
